# Identifying the origin of local flexibility in a carbohydrate polymer

**DOI:** 10.1073/pnas.2102168118

**Published:** 2021-05-31

**Authors:** Kelvin Anggara, Yuntao Zhu, Giulio Fittolani, Yang Yu, Theodore Tyrikos-Ergas, Martina Delbianco, Stephan Rauschenbach, Sabine Abb, Peter H. Seeberger, Klaus Kern

**Affiliations:** ^a^Nanoscale Science Department, Max Planck Institute for Solid State Research, 70569 Stuttgart, Germany;; ^b^Biomolecular Systems Department, Max Planck Institute of Colloids and Interfaces, 14476 Potsdam, Germany;; ^c^Department of Chemistry and Biochemistry, Freie Universität Berlin, 14195 Berlin, Germany;; ^d^Chemistry Research Laboratory, Department of Chemistry, University of Oxford, OX1 3TA Oxford, United Kingdom;; ^e^Institut de Physique, École Polytechnique Fédérale de Lausanne, CH-1015 Lausanne, Switzerland

**Keywords:** structure–property relationship, glycan flexibility, automated synthesis

## Abstract

Correlating the structures and properties of a polymer to its monomer sequence is key to understanding how its higher hierarchy structures are formed and how its macroscopic material properties emerge. Carbohydrate polymers, such as cellulose and chitin, are the most abundant materials found in nature whose structures and properties have been characterized only at the submicrometer level. Here, by imaging single-cellulose chains at the nanoscale, we determine the structure and local flexibility of cellulose as a function of its sequence (primary structure) and conformation (secondary structure). Changing the primary structure by chemical substitutions and geometrical variations in the secondary structure allow the chain flexibility to be engineered at the single-linkage level. Tuning local flexibility opens opportunities for the bottom-up design of carbohydrate materials.

## Significance

The monomer sequence dictates the structure and properties of natural polymers. Such a structure–property relationship is well known for polypeptides and polynucleotides but not for polysaccharides, the most abundant biopolymers on Earth. Here, we establish the structure–property relationship for a polysaccharide at the atomic level by determining molecular flexibility of carbohydrate chains with defined sequences. The chain flexibility can be engineered one linkage at a time by chemical substitution and conformation change, highlighting how the primary and secondary structures of a carbohydrate dictate its flexibility—a critical observable in the de novo design of carbohydrate materials. Our approach can be extended to establish the structure–property relationship at the atomic level of any molecule that can be electrosprayed.

Natural polymers adopt a multitude of three-dimensional structures that enable a wide range of functions (1). Polynucleotides store and transfer genetic information; polypeptides function as catalysts and structural materials; and polysaccharides play important roles in cellular structure (2–6), recognition (5), and energy storage (7). The properties of these polymers depend on their structures at various hierarchies: sequence (primary structure), local conformation (secondary structure), and global conformation (tertiary structure).

Automated solid-phase techniques provide access to these polymers with full sequence control (8–12). The correlation between the sequence, the higher hierarchy structures, and the resulting properties is relatively well established for polynucleotides (13, 14) and polypeptides (15, 16), while comparatively little is known for polysaccharides (17). Unlike polypeptides and polynucleotides, polysaccharides are based on monosaccharide building blocks that can form multiple linkages with different configurations (e.g., α- or β-linkages) leading to extremely diverse linear or branched polymers. This complexity is exacerbated by the flexibility of polysaccharides that renders structural characterization by ensemble-averaged techniques challenging (17). Imaging single-polysaccharide molecules using atomic force microscopy has revealed the morphology and properties of polysaccharides at mesoscopic, submicrometer scale (18–22). However, imaging at such length scales precludes the observation of individual monosaccharide subunits required to correlate the polysaccharide sequence to its molecular structure and flexibility, the key determinants of its macroscopic functions and properties (23).

Imaging polysaccharides at subnanometer resolution by combining scanning tunnelling microscopy (STM) and electrospray ion-beam deposition (ES-IBD) (24, 25) allows for the observation of their monosaccharide subunits to reveal their connectivity (26–28) and conformation space (29). Here, we use this technique to correlate the local flexibility of an oligosaccharide chain to its sequence and conformation, the lowest two structural hierarchies. By examining the local freedom of the chain as a function of its primary and secondary structures, we address how low-hierarchy structural motifs affect local oligosaccharide flexibility—an insight critical to the bottom-up design of carbohydrate materials (30).

**Fig. 1.**
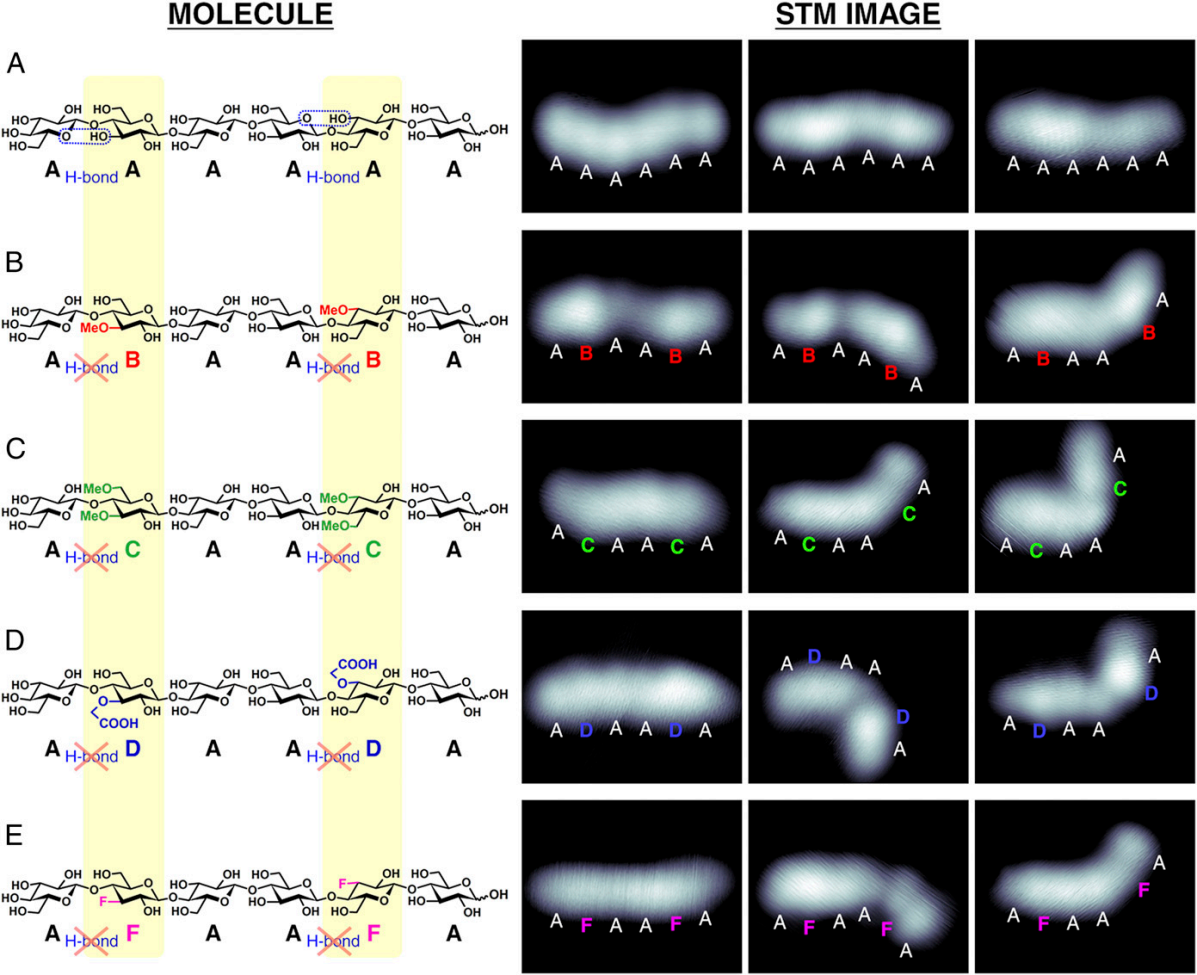
STM images of cellohexaose (AAAAAA) and its analogs (AXAAXA). Structures and STM images of cellohexaose (*A*) and its substituted analogs (*B*–*E*). Cellohexaose contains six Glcs (labeled as A; colored black) linked via β-1,4–glycosidic bonds. The cellohexaose analogs contain two substituted Glcs, as the second and the fifth residues from the nonreducing end, that have a single methoxy (–OCH_3_) at C(3) (labeled as B; colored red), two methoxy groups at C(3) and C(6) (labeled as C; colored green), a single carboxymethoxy (–OCH_2_COOH) at C(3) (labeled as D; colored blue), and a single fluorine (–F) at C(3) (labeled as F; colored purple).

We elucidate the origin of local flexibility in cellulose, the most abundant polymer in nature, composed of glucose (Glc) units linked by β-1,4–linkages (31–33). Unveiling what affects the flexibility of cellulose chains is important because it gives rise to amorphous domains in cellulose materials (34–37) that change the mechanical performance and the enzyme digestibility of cellulose (38). Cellohexaose, a Glc hexasaccharide (Fig. 1*A*), was used as a model for a single-cellulose chain as it has been shown to resemble the cellulose polymer behavior (12). Modified analogs prepared by Automated Glycan Assembly (AGA) (11, 12) were designed to manipulate particular intramolecular interactions responsible for cellulose flexibility. Cellohexaose, ionized as a singly deprotonated ion in the gas phase ([M-H]^−1^) was deposited on a Cu(100) surface held at 120 K by ES-IBD (24) (*Materials and Methods*). The ions were landed with 5-eV energy, well suited to access diverse conformation states of the molecule without inducing any chemical change in the molecule (29). The resulting cellohexaose observed in various conformation states allowed its mechanical flexibility (defined by the variance in the geometrical bending between two residues) to be quantified for every intermonomer linkage. The observed dependence of local flexibility on the oligosaccharide sequence and conformation thus exemplifies how primary and secondary structures tune the local mechanical flexibility of a carbohydrate polymer.

The effect of the primary structure on the chain flexibility was explored using sequence-defined cellohexaose analogs (Fig. 1). Cellohexaose, AAAAAA (Fig. 1*A*), was compared with its substituted analogs, ABAABA, ACAACA, ADAADA, and AFAAFA (written from the nonreducing end) (Fig. 1 *B*–*E*), where A is Glc, B is Glc methylated at OH(3), C is Glc methylated at OH(3) and OH(6), D is Glc carboxymethylated at OH(3), and F is Glc deoxyfluorinated at C(3). These substitutions are designed to alter the intramolecular hydrogen bonding between the first and the second as well as between the fourth and fifth Glc units (Fig. 1). These functional groups also affect the local steric environment (i.e., the bulky carboxymethyl group) (Fig. 1*D*) and the local electronic properties (i.e., the electronegative fluorine group) (Fig. 1*E*). When compared with the unsubstituted parent cellohexaose, these modified cellohexaoses exhibit different aggregation behavior and are more water soluble (12).

All cellohexaose derivatives adsorbed on the surface were imaged with STM at 11 K (Fig. 1). The oligosaccharides were deposited as singly deprotonated species and were computed to adsorb on the surface via a single covalent RO–Cu bond, except for ADAADA which was deposited as doubly deprotonated species and was computed to adsorb on the surface via two covalent RCOO–Cu bonds (R = sugar chain). All cellohexaoses appear as chains containing six protrusions corresponding to the six constituent Glcs. The unmodified cellohexaose chains (Fig. 1*A*) mainly adopt a straight geometry, while the substituted cellohexaoses (Fig. 1 *B*–*E*) adopt both straight- and bent-chain geometries. Chemical substitution thus increases the geometrical freedom of the cellulose chain, consistent with the reported macroscopic properties (12).

Large-chain bending between neighboring Glc units is observed exclusively for the substituted cellohexaose (Fig. 1). The large, localized bending reveals the substitution site and allows for the nonreducing and the reducing ends of the chain to be identified. These chains are understood to bend along the surface plane via the glycosidic linkage without significant tilting of the pyranose ring that remains parallel to the surface (illustrated in *SI Appendix*, Fig. S1), as indicated by the ∼2.0-Å height of every Glc (29).

**Fig. 2.**
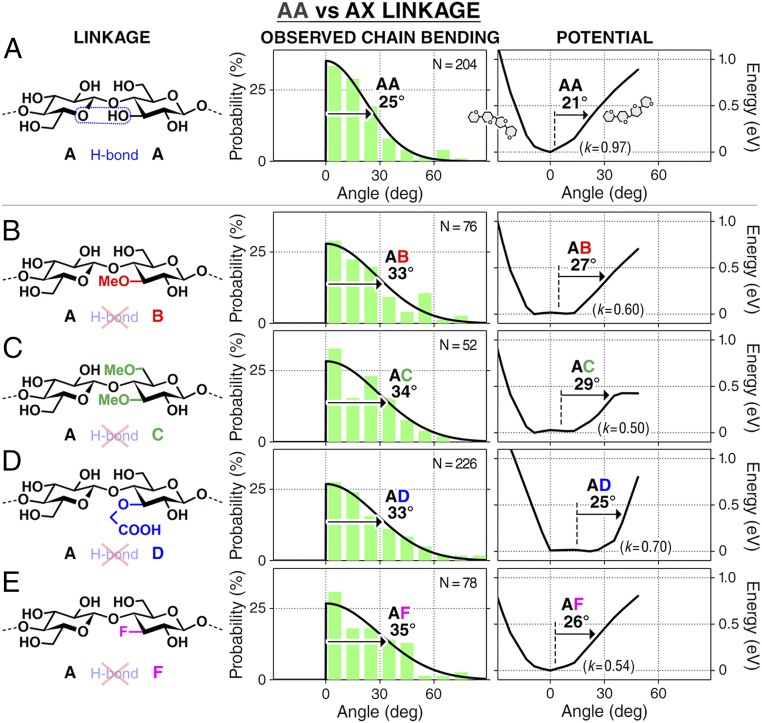
Bending flexibility of AA linkage and substituted AX linkages. Chain bending (Fig. 1) is quantified as an angle formed between two neighboring Glcs (*Materials and Methods*). The results are given in *A* for AA, in *B* for AB, in *C* for AC, in *D* for AD, and in *E* for AF, showing that AX (where X = B, C, D, F) has a higher conformational freedom than AA. The angle distributions (bin size: 10°) are fitted with a Gaussian (solid line) shown with its half-width half-maximum. The computed potential energy curves are shown with the half-width at 0.4 eV and fitted with a parabola to estimate its stiffness (*k*; in millielectronvolts per degree^2^).

The bending angle measured for AA and AX linkages (Fig. 2; *Materials and Methods* has analysis details) shows that, while both AA and AX prefer the straight, unbent geometry, AX displays a greater variation of bending angles than AA. AX angular distribution is consistently ∼10° wider than that for AA, indicating that AX has a greater conformational freedom than AA. This increased bending flexibility results from the absence of the intramolecular hydrogen bonding between OH(3) and O(5) of the neighboring residue. Methylation of OH(6), in addition to methylation of OH(3), results in similar flexibility (Fig. 2 *B* and *C*), suggesting the greater importance of OH(3) in determining the bending flexibility. Steric effects were found to be negligible since AD displayed similar flexibility to other less bulky AX linkages.

Density functional theory (DFT) calculations support the observations, showing that substitution of OH(3) decreases the linkage stiffness by up to ∼40% (Fig. 2). Replacing OH(3) with other functional groups weakens the interglucose interactions by replacing the OH(3)··O(5) hydrogen bond with weak Van der Waals interactions. The similar flexibility between AB and AC linkages is attributed to the similar strength of the interglucose OH(2)··OH(6) hydrogen bond in AB (Fig. 2*B*) and the OH(2)··OMe(6) hydrogen bond in AC (Fig. 2*C*). The negligible steric effect in AD is attributed to the positional and rotational freedom of the bulky moiety that prevents any “steric clashes” and diminishes the contribution of steric repulsion in the potential energy curve. Comparing the potential landscape in the gas phase and on the surface shows that the stiffness of the adsorbed cellohexaoses is primarily dictated by their intramolecular interactions instead of molecule–surface interactions (*SI Appendix*, Fig. S2). Primary structure alteration by chemical substitution modifies the interglucose hydrogen bonds and enables chain flexibility to be locally engineered at the single-linkage level.

**Fig. 3.**
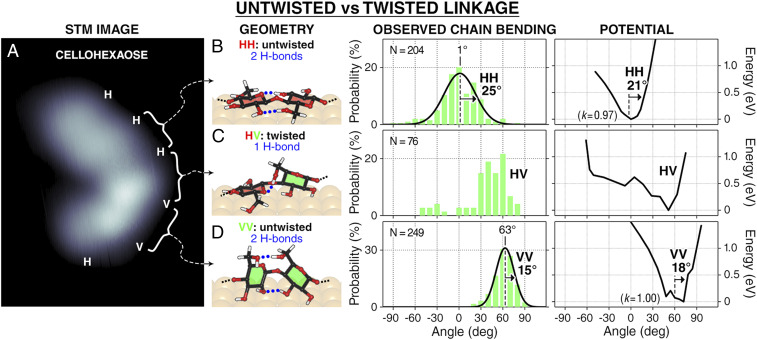
Bending flexibility of untwisted and twisted AA linkages. (*A*) STM image of a cellohexaose containing two types of AA linkages: untwisted (HH and VV) and twisted (HV and VH; from the nonreducing end). The measured bending angles and the computed potential curve are given in *B* for HH, in *C* for HV, and in *D* for VV, showing that the twisted linkage (HV) is more flexible than the untwisted ones (HH and VV). In the molecular structures, interunit hydrogen bonds are given as dotted blue lines, and the pyranose rings are colored red for the horizontal ring (H) and green for vertical (V). The angle distributions (bin size: 10°) are fitted with a Gaussian distribution (solid line) labeled with its peak and half-width half-maximum. The computed potential curves are labeled with its half-width at 0.4 eV and fitted with a parabola to estimate its stiffness (*k*; in millielectronvolts per degree^2^).

**Fig. 4.**
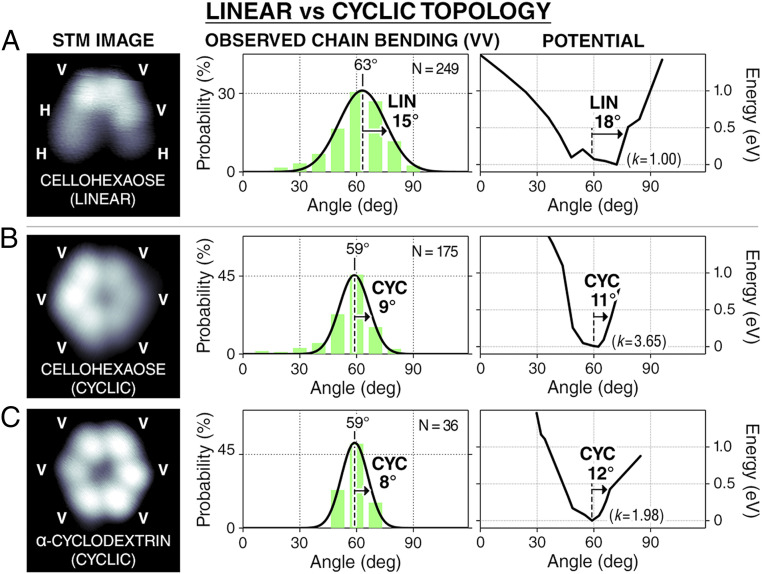
Bending flexibility of AA linkage in linear (LIN) and cyclic (CYC) chains. STM image, measured bending angle distribution, and computed potential of AA linkage are given in *A* for a linear cellohexaose conformer and in *B* for a cyclic cellohexaose conformer, showing that chain flexibility is reduced in conformations with cyclic topology. The same data are given in *C* for α-cyclodextrin that is locked in a conformation with cyclic topology. The measured angles (bin size: 10°) are each fitted with a Gaussian distribution (solid line) labeled with its peak and half-width half-maximum. The computed potentials are each labeled with its half-width at 0.4 eV and fitted with a parabola to estimate its stiffness (*k*; in millielectronvolts per degree^2^).

We subsequently investigate how molecular conformation (secondary structure) affects the local bending flexibility. We define the local secondary structure as the geometry formed between two Glcs, here exemplified by the local twisting of the chain (Fig. 3). The global secondary structure is defined as the overall geometry formed by all Glcs in the chain, here exemplified by the linear and cyclic topologies of the chain (Fig. 4).

The effect of local secondary structure on chain flexibility is exemplified by the bending flexibility of twisted and untwisted linkages in a cellohexaose chain (Fig. 3*A*). The untwisted and twisted linkages are present due to the Glc units observed in two geometries, H or V (Fig. 3), distinguished by their heights (*h*). H (*h* ∼ 2.0 Å) is a Glc with its pyranose ring parallel to the surface, while V (*h* ∼ 2.5 Å) has its ring perpendicular to the surface (29). These lead to HH and VV as untwisted linkages and HV and VH (written from nonreducing end) as twisted linkages.

The twisted linkage is more flexible than the untwisted one, as shown by the unimodal bending angles for the untwisted linkage (HH and VV in Fig. 3 *B* and *D*, respectively) and the multimodal distribution for the twisted linkage (HV in Fig. 3*C*). DFT calculations attribute the increased bending flexibility to the reduction of accessible interunit hydrogen bonds from two to one. Linkage twisting increases the distance between the hydrogen-bonded pair, which weakens the interaction between Glc units and increases the flexibility at the twisting point. The increase in local chain flexibility conferred by chain twisting shows how local secondary structures affect chain flexibility.

The effect of the global secondary structure on the local chain flexibility was examined by comparing the local bending flexibility of cellohexaose chains possessing different topologies. Cellohexaose can adopt either linear (Figs. 3*A* and 4*A*) or cyclic topology (Fig. 4*B*), the latter characterized by the presence of a circular, head-to-tail hydrogen bond network (29). The cyclic conformation of cellohexaose is enabled by the head-to-tail chain folding from the 60° chain bending of the VV linkage. The VV segment in the cyclic chain is stiffer than in the linear chain since the bending angle distribution for the cyclic chain is 6° narrower than that for the linear chain. The observation is corroborated by DFT calculations that show that the VV linkage in the cyclic chain is about three times stiffer than that in the linear chain.

To characterize the degree of chain stiffening due to the linear-to-cyclic chain folding, we compare the flexibility of the cyclic cellohexaose and α-cyclodextrin (an α-1,4–linked hexaglucose covalently locked in cyclic conformation). The α-cyclodextrin provides the referential local flexibility for a cyclic oligosaccharide chain. Strikingly, the local flexibility in α-cyclodextrin was found to be identical to that in the cyclic cellohexaose, as evidenced by the similar width of the bending angle distribution and the computed potentials (Fig. 4 *B* and *C*). The similar stiffness indicates that the folding-induced stiffening in cellohexaose is a general topological effect unaffected by the type of the interactions that give the cyclic conformation (noncovalent hydrogen bond in cellohexaose vs. covalent bond in α-cyclodextrin). The folding-induced stiffening is the result of the creation of a circular spring network that restricts the motion of Glc units and reduces their conformational freedom. The folding-induced stiffening reported here provides a mechanism by which carbohydrate structures can be made rigid. The dependence of the local chain flexibility on the chain topology shows how global secondary structures modify local flexibility.

Using cellulose as an example, we have quantified the local flexibility of a carbohydrate polymer and identified structural factors that determine its flexibility. Modification of the carbohydrate primary structure by chemical substitution alters the mechanical flexibility at the single-linkage level. Changing secondary structure by chain twisting and folding provides additional means to modify the flexibility of each linkage. Control of these structural variables enables tuning of polysaccharide flexibility at every linkage as a basis for designing and engineering carbohydrate materials (30). Our general approach to identify structural factors affecting the flexibility of a specific molecular degrees of freedom in a supramolecular system should aid the design of materials and molecular machines (39) and the understanding of biomolecular dynamics.

## Materials and Methods

### Experiment.

The experiment was conducted at 11 K using a Scanning Tunneling Microscope (Omicron Fermi SPM) in ultrahigh vacuum (P ∼ 10^−10^ mbar). The Cu(100) surface was cleaned by repeated sputtering with Ar ions at 1 keV and annealing at ∼600 K. The molecules under study, cellohexaose (AAAAAA) and its derivatives (ABAABA, ACAACA, ADAADA, and AFAAFA), were synthesized using the AGA method reported previously (12); α-cyclodextrin (98%) was purchased from Sigma-Aldrich. The molecules were prepared as a beam of negative molecular ions using the ES-IBD apparatus (24) and were deposited at normal incident angle with 5.0-eV landing energy on a clean Cu(100) surface held at ∼120 K. The molecular ions in the beam, prior to their deposition on surface, were selected based on its mass-to-charge ratio (*m/z*) measured by a home-built time-of-flight mass spectrometer in our ES-IBD apparatus (i.e., [M-H]^−1^ was used for AAAAAA [*m/z* = 988], ABAABA [*m/z* = 1,017], ACAACA [*m/z* = 1,045], AFAAFA [*m/z* = 993], and α-cyclodextrin [*m/z* = 971], while [M-2H]^−2^ was used for ADAADA [*m/z* = 552]). The spray solution used was 640 μL of 10^−5^ mol/L sugar solution in 4:5 water:ethanol (vol/vol) solution combined with a 10-μL aliquot of 15% (vol/vol) aqueous NH_3_. The molecules on the surface were imaged at constant-current mode with a set point of ∼1 pA and surface bias of +0.3 V. The positions of the Glc units in the sugar chain were measured using the WSxM software (40). The chain bending angle formed at each glycosidic linkage was obtained by taking the difference of the chain tangent at neighboring Glc units. The width of the resulting angular distribution was used to compare different flexibilities observed for different linkages.

### Theory.

DFT calculations with a plane-wave basis set were performed using the Vienna Ab-Initio Simulation Package (version 5.4.4) (41, 42). The calculations used the projected augmented wave, generalized gradient approximation (43, 44), Perdew–Bruke–Ernzerhof functional (45), and the third version of Grimme’s semiempirical Van der Waals correction (known as DFT-D3) (46). The calculations were limited to the gamma point and used 400-eV cutoff energy. The Cu(100) surface was represented by a (11 × 6) slab that consisted of 198 Cu atoms in three layers with a vacuum gap of 21 Å. The potential energy curves in Figs. 2 and 3 were computed by 1) preparing several initial structures with variously bent glycosidic bond between the second and third Glc units in cellotetraose (AAAA) and modified cellotetraose (AAXA, written from the nonreducing end) and 2) relaxing these structures while keeping constant the *x* and *y* coordinates of C1 and C4 for every pyranose rings. The relaxation was performed with the bottom layer of the Cu slab frozen, until the residual force on each atom was <0.01 eV/Å to give potential curves and structures (*SI Appendix*, Fig. S1 has an example). The potential energy curves in Fig. 4 were obtained with the same method but with a different Cu slab [i.e., three layers of (10 × 8) slab] to accommodate the larger cyclic cellohexaose and α-cyclodextrin. The computed energy landscape shows, for every linkage, a potential well whose width indicates its flexibility (i.e., the wider the potential, the larger its flexibility). To aid the comparison between different potential wells, we have estimated the stiffness (*k*) associated with each potential well by assuming the linkage as a harmonic oscillator and fitting the computed potential with a parabola. The molecular structures were visualized using the VESTA software (47).

## Supplementary Material

Supplementary File

## Data Availability

All data needed to evaluate the conclusion in the paper are present in the paper or *SI Appendix*.
