# Supplementary material for: Identifying the origin of local flexibility in a carbohydrate polymer
Source: Proc Natl Acad Sci U S A. 2021 May 31;118(23):e2102168118. doi: 10.1073/pnas.2102168118 (PMC8201824; doi:10.1073/pnas.2102168118)
Supplement: Supplementary File [file pnas.2102168118.sapp.pdf]

PNAS
www.pnas.org

Supplementary Information for
Identifying the Origin of Local Flexibility in a Carbohydrate Polymer

Kelvin Anggara, Yuntao Zhu, Giulio Fittolani, Yang Yu, Theodore Tyrikos-Ergas, Martina Delbianco, Stephan Rauschenbach, Sabine Abb, Peter H. Seeberger, Klaus Kern

Corresponding Authors: Kelvin Anggara, Peter H. Seeberger, Klaus Kern
Email: k.anggara@fkf.mpg.de, peter.seeberger@mpikg.mpg.de, k.kern@fkf.mpg.de

This PDF file includes:

Figures S1 to S2

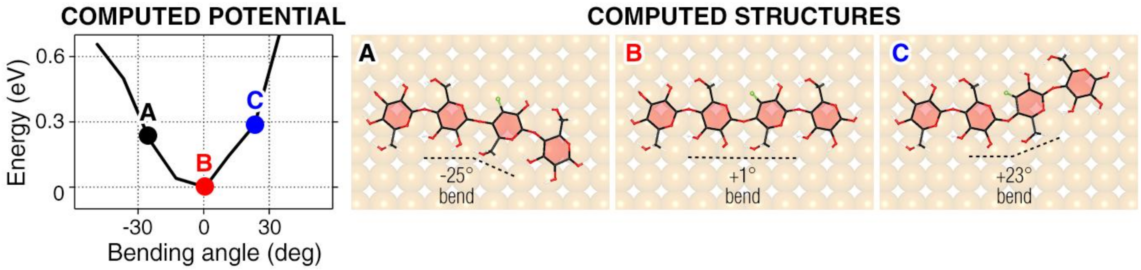

Fig. S1. An example of a computed potential energy curve and its corresponding structures. The computed potential energies plotted as a function of bending angle of an AAXA cellotetraose adsorbed on Cu(100) surface. The bending angle is varied by bending along the surface-plane the linkage between the second and the third glucose i.e. the AA-XA linkage (written from the non-reducing end). Three structures are shown without the axial H-atoms for clarity, and with the pyranose ring colored in red.

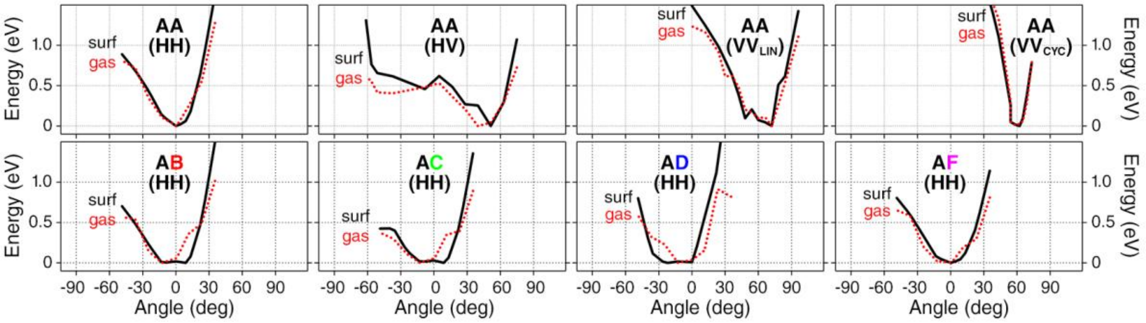

Fig. S2. Comparing the potential energy surface for cellohexaose on surface and in gas phase. The similar potential landscape between gas-phase cellohexaose (red dotted line) and adsorbed cellohexaose (black solid line) shows that the potential landscape of the adsorbed cellohexaose is largely determined by its intramolecular interactions instead of its molecule-surface interaction. The gas-phase potential landscape is obtained by performing single-point energy calculation for every cellohexaose geometries without the surface.
